# Enhanced Electroacoustic Tomography with Supervised Learning for Real‐time Electroporation Monitoring

**DOI:** 10.1002/pro6.1242

**Published:** 2024-09-22

**Authors:** Zhuoran Jiang, Yifei Xu, Leshan Sun, Shreyas Srinivasan, Q. Jackie Wu, Liangzhong Xiang, Lei Ren

**Affiliations:** ^1^ Stanford University Stanford USA; ^2^ University of California Irvine USA; ^3^ Duke University Medical Center Durham USA; ^4^ University of Maryland School of Medicine Baltimore USA

**Keywords:** electroacoustic tomography, electroporation, interventional therapy, limited‐angle reconstruction, supervised learning

## Abstract

**Background:**

Nanosecond pulsed electric fields (nsPEF)‐based electroporation is a new therapy modality potentially synergized with radiation therapy to improve treatment outcomes. To verify its treatment accuracy intraoperatively, electroacoustic tomography (EAT) has been developed to monitor in‐vivo electric energy deposition by detecting ultrasound signals generated by nsPEFs in real‐time. However, utility of EAT is limited by image distortions due to the limited‐angle view of ultrasound transducers.

**Methods:**

This study proposed a supervised learning‐based workflow to address the ill‐conditioning in EAT reconstruction. Electroacoustic signals were detected by a linear array and initially reconstructed into EAT images, which were then fed into a deep learning model for distortion correction. In this study, 56 distinct electroacoustic data sets from nsPEFs of different intensities and geometries were collected experimentally, avoiding simulation‐to‐real‐world variations. Forty‐six data were used for model training and 10 for testing. The model was trained using supervised learning, enabled by a custom rotating platform to acquire paired full‐view and single‐view signals for the same electric field.

**Results:**

The proposed method considerably improved the image quality of linear array‐based EAT, generating pressure maps with accurate and clear structures. Quantitatively, the enhanced single‐view images achieved a low‐intensity error (RMSE: 0.018), high signal‐to‐noise ratio (PSNR: 35.15), and high structural similarity (SSIM: 0.942) compared to the reference full‐view images.

**Conclusions:**

This study represented a pioneering stride in achieving high‐quality EAT using a single linear array in an experimental environment, which improves EAT's utility in real‐time monitoring for nsPEF‐based electroporation therapy.

## Graphical abstract

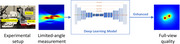

## 1 INTRODUCTION

The use of electricity for human therapy has a long history, dating back to the 18th century and continuing to develop over the centuries.^1^, ^2^ Currently, electric fields are being utilized in a wide range of biomedical and clinical applications. Low‐intensity electric fields have shown promise in wound healing,^3^ tumor treatment,^4^ and tissue engineering.^5^ Meanwhile, high‐intensity pulsed electric fields can induce cell membrane perforation, allowing for electroporation,^6^, ^7^ a technique utilized in DNA transfection,^7^, ^8^ drug delivery,^7^ electrochemical therapy,^9^ and tissue ablation.^10^ Recently, research has been conducted on electroporation using nanosecond pulsed electric fields (nsPEF). This body of work has demonstrated nsPEF's effects on cell membranes and intracellular protein structures,^11^ suggesting their potential to be synergized with radiation therapy to improve treatment outcomes. Serša et al.^12^ found that using electroporation to deliver cisplatin into tumor cells enhances the radiosensitizing effect of the drug. In addition, a study by Yadollahpour et al.^13^ demonstrated that pre‐radiotherapy electroporation significantly increases sensitivity in human intestinal colon cancer HT‐29 cells. However, in these nsPEF‐based treatments, preoperative planning is typically done through numerical simulation,^14^ and postoperative evaluation is conducted using MRI, ultrasound, or other methods.^15^ It is highly desirable to have an intraoperative imaging method that can monitor the electric field energy deposition in real time to verify the precision of the treatment.

To meet this clinical need, we have proposed electroacoustic tomography (EAT), an acoustic‐based imaging method that can reconstruct real‐time energy distribution of an electric field in deep tissues.^16^ EAT was a label‐free, radiation‐free, and non‐invasive imaging method. It utilized clinically common linear ultrasound probes and can be combined with off‐the‐shelf ultrasound imaging equipment for dual‐mode hybrid imaging.^17^, ^18^ This technique revealed the electrical field in tissues by detecting the ultrasound signals arising from the nsPEF energy absorption in tissues.^18^ As a result, it required a full‐view acquisition of electroacoustic signals to reconstruct images without distortions. However, clinically widely used ultrasound detectors, such as linear or convex arrays, have limited acquisition angles, which resulted in severe image distortions and limited EAT's clinical utilities. Methods to alleviate the distortions, such as adjusting the projection angle through rotation and displacement or utilizing multiple probes for concurrent imaging, often necessitate supplementary equipment and elevate the costs, making clinical translation problematic. Thus, there is an urgent clinical need to develop algorithms to reconstruct high‐quality EAT images from the limited‐angle measurement

Essentially, limited‐angle image reconstruction is an ill‐conditioned inverse problem. Compressed sensing (CS)‐based methods have been developed to reconstruct under‐sampled images by exploiting their sparsity in certain domains. But they have limited effectiveness in correcting the significant geometric distortions^19^, ^20^, ^21^ seen in linear array‐based EAT. In recent years, deep learning has revolutionized various image‐related tasks.^22^, ^23^, ^24^, ^25^, ^26^, ^27^, ^28^ In particular, it has shown superior performance in reconstructing images using limited‐angle measurements. Huang et al.^29^ proposed a deep learning model for CT image reconstruction from limited‐angle measurements, and achieved considerably improved root‐mean‐squared‐errors (RMSE). Shen et al.^30^ demonstrated the feasibility of deep learning to reconstruct computed tomography (CT) images from a single projection using a patient‐specific strategy. Our previous studies^20^, ^31^, ^32^ showed the effectiveness of deep learning in generating high‐quality images for limited‐angle cone‐beam CT (CBCT), matrix array‐based protoacoustic imaging, and single Compton camera‐based prompt gamma imaging.

Considering the advantages of deep learning in restoring volumetric information from limited‐angle acquisitions, we aim to explore its feasibility in enhancing the image quality of linear array‐based EAT. In this study, we developed a modified U‐Net^33^ to correct the distortions in the single‐view EAT images.

For model training, the supervised learning strategy was adopted due to its clear objective measurement and superior accuracy for predictive tasks. However, this strategy requires well‐labeled data, posing challenges in many medical image‐related applications. Data simulation has been a widely used solution, but it can cause performance degradation in deep learning models, depending on the simulation‐to‐real‐world variations. To bridge this gap, in this study, we designed a custom rotating platform to acquire paired full‐view and single‐view signals for the same electric field. The proposed method was trained and tested using experimental data, further confirming its utility in real‐world applications.

To our knowledge, this is the first time accurate pressure maps are generated from electroacoustic signals measured by a linear array in an experimental environment, which considerably improves the EAT's clinical utility in the real‐time monitoring of electroporation therapy.

## 2 METHODS

### 2.1 Problem formulation

Let $x\;\in \;R^{I\times J}$ be the real‐valued limited‐angle reconstructed electroacoustic images with dimensions I×J voxels, and $y\;\in \;R^{I\times J}$ be the corresponding full‐view reconstructed images. The task can be formulated as finding an image‐enhancing pattern f between the single‐view image x and the corresponding full‐view image y so that

$f\;=\;\underset{f}{\mathrm{argmin}}\left({∥f\left(x\right)-y∥}_{2}^{2}\right)$.

### 2.2 Deep learning‐based EAT image enhancement

**FIGURE 1:**
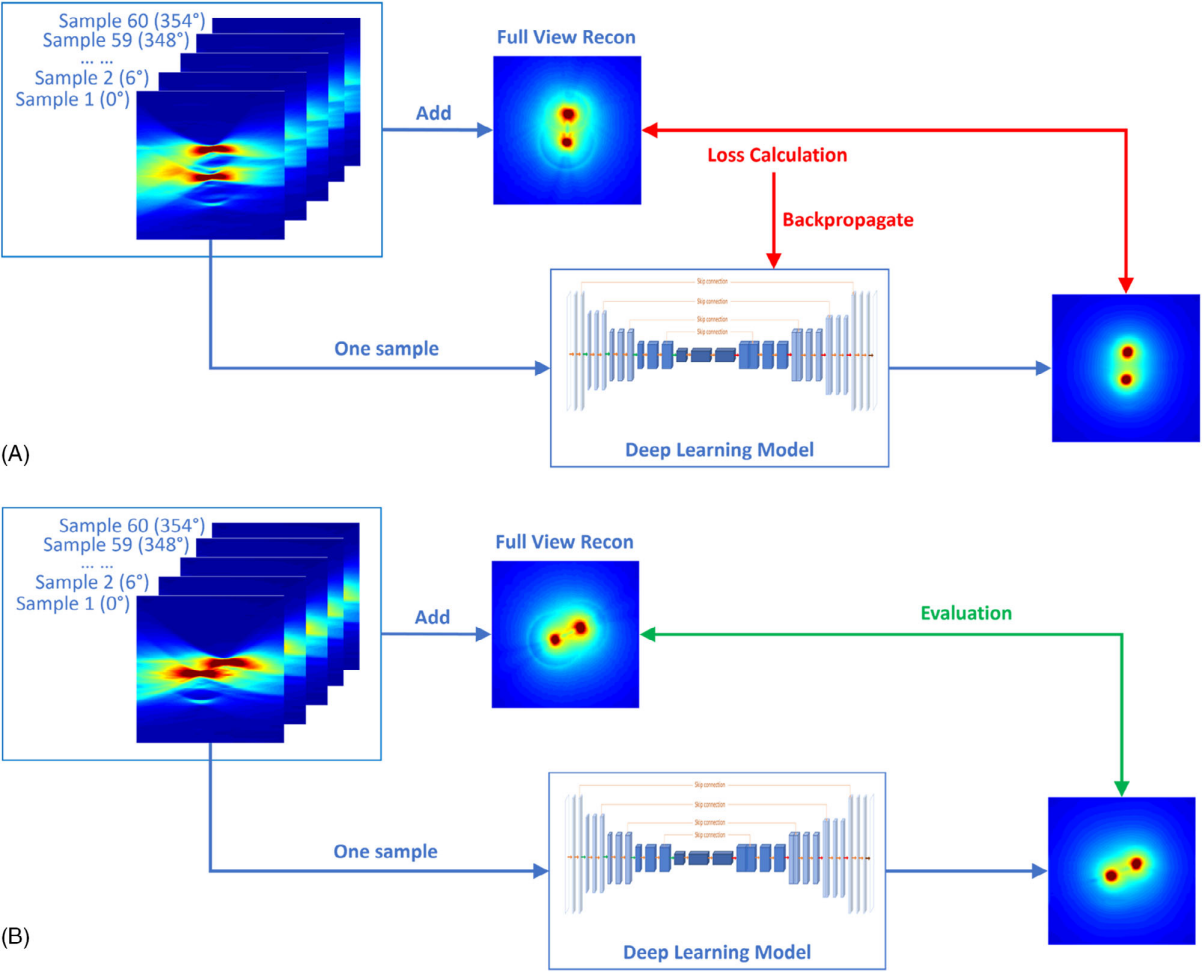
Overall workflow for deep learning‐enhanced electroacoustic tomography (EAT) using a linear array. (A) is the model training process. (B) is model testing process. During training, the single‐view image was fed into the model, which was trained to enhance image matching with the ground truth full‐view image. Loss between the enhanced and the ground truth images was calculated to update the model weights. During testing, the model's prediction was compared to the ground truth for evaluation. The model was trained and tested using different datasets.

Figure 1 shows the overall workflow of the proposed deep learning‐based EAT image enhancement. Single‐view images were reconstructed from the electroacoustic signals measured by a linear array using the back‐projecting algorithm, and were then fed into the deep learning model to correct the distortions. During training, model's weights were optimized by minimizing the dissimilarity between the enhanced and the ground truth images. During testing, single‐view images were enhanced by the trained model, and then were compared to the ground truth images for evaluation. Note that the model was trained and tested using different datasets.

In this study, the full‐view images were reconstructed using electroacoustic signals measured by the linear array at all angles and were used as the ground truth.

**FIGURE 2:**
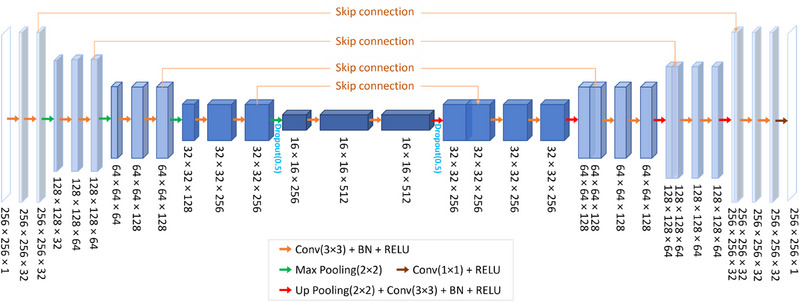
Structure of the deep learning model (modified U‐Net) implemented in this study. The model takes the single‐view image as input, and generates enhanced image. Numbers in the figure indicate the feature/input/output dimensions

Figure 2 shows the detailed structure of the deep learning model developed. Specifically, a multi‐scale U‐Net structure was used for the image enhancement due to its effectiveness in addressing image features. In this study, we modified the original U‐Net in several aspects. First, batch normalization layers were used to normalize the features extracted by the preceding convolutional layers. This normalization technique has been well‐established in stabilizing and accelerating the training process with higher learning rates. Second, dropout layers with a 0.5 dropout rate were used in the U‐bottom to improve the model's generalizing abilities and to avoid overfitting. Third, structural similarity was used in the training process to supervise a more accurate and realistic output.

## 3 EXPERIMENTAL DESIGN

### 3.1 Data acquisition

#### 3.1.1 Experimental setup

**FIGURE 3:**
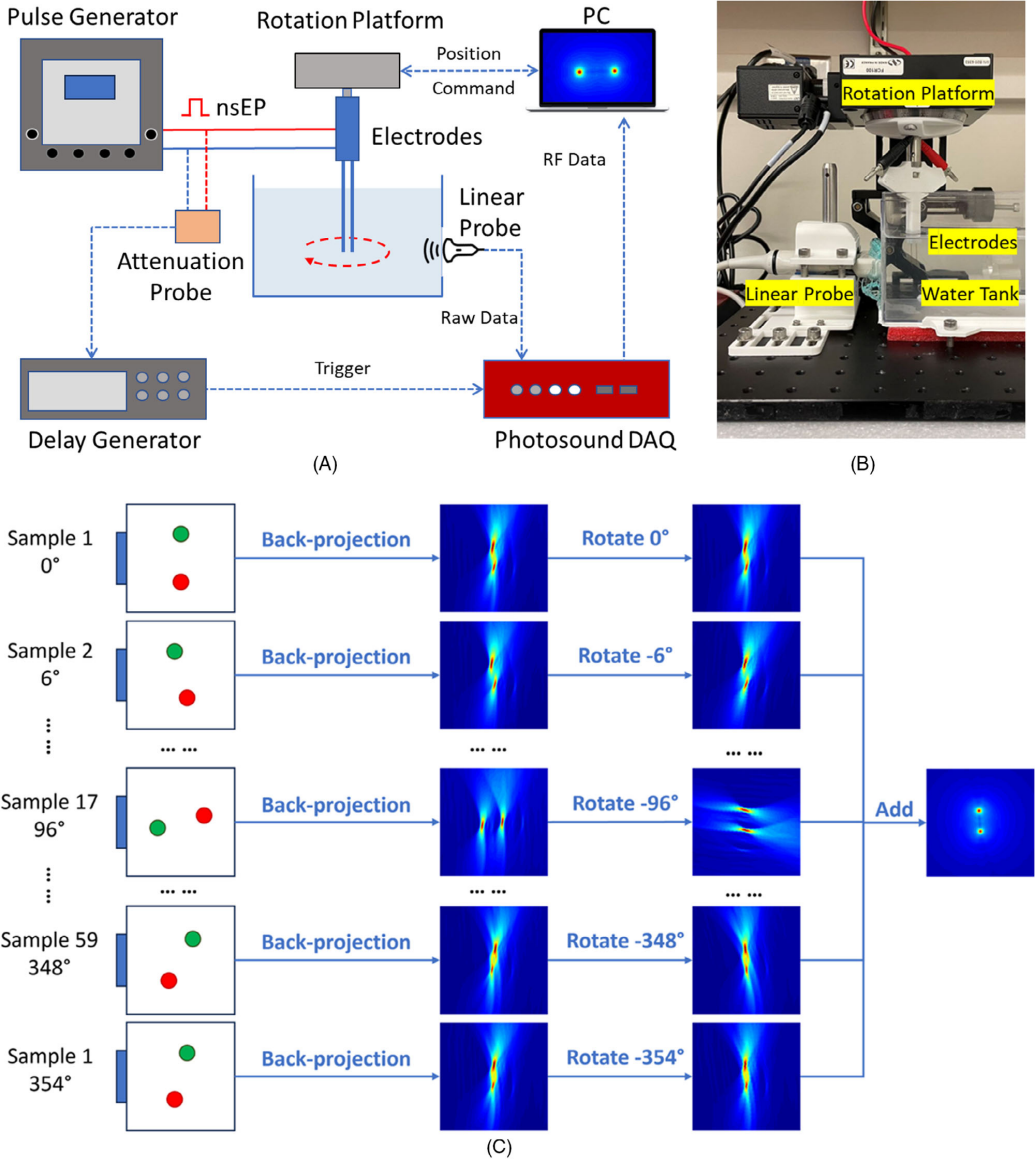
Experiment setup. (A) is an illustration. (B) is a photo of the experiment setup in this study. (C) indicates the full‐view image acquisition and reconstruction. More details can be found in section 3.1.

In this study, a custom nanosecond electrical pulse generator (VilniusTECH, Vilnius, Lithuania) was used to produce electrical pulses with durations of up to 100 nanoseconds, adjustable amplitudes ranging from 0 to 2 kV, and variable repetition frequencies from 1 Hz to 1 MHz. The electrical pulses were delivered to objects via tungsten electrodes (57400, A&M System, USA), which were fixed to a rotating platform (FCR100, Newport Corporation, USA) using a 3D‐printed holder. High‐voltage pulses were detected by a high‐voltage probe (P4250, Keysight Technologies, USA), and they were subsequently attenuated and fed into a delay generator (DG535, STANFORD RESEARCH, USA) to output a standard TTL trigger signal for data acquisition. The ultrasound signals induced by nsPEF were detected by a clinical 128‐channel linear‐array ultrasound probe (L12‐5L60N, Telemed Medical Systems, Italy), and were digitized in parallel by a 128‐channel data acquisition (DAQ) system (Photosound, USA). The system has a sampling rate of 40 MHz and a depth of 12 bits to ensure the accuracy of the acquired electroacoustic signals. The ultrasound transducer was placed outside the tank to minimize the influence of the high‐voltage pulsed electric field on the piezoelectric ultrasound probe. A hole was cut in the side of the water tank and covered by a polyethylene film to enable the coupling of the transducer through the ultrasound gel. The entire system was controlled by a MATLAB program that automatically released the electrical pulses, rotated the electrodes, and stored the ultrasound signals. An illustration of the experimental setup is shown in Figure 3.

#### 3.1.2 Dataset generation

In this study, 56 data sets were acquired using various arrangements of electrodes (2 electrodes, various distances and angles relative to the linear array) with different voltages (1200 – 2400 Volts/cm). Data sets were acquired with electrodes placed at different distances and angles relative to the linear array, resulting in substantially different responses in the detector.

For each data set, we measured signals from 60 equally distributed views over 360° using a linear array. As shown in Figure 3(C), each single‐view image was reconstructed from the signals measured in the corresponding view using the back‐projection algorithm, and the full‐view image was reconstructed by rotating and averaging all single‐view images together.

### 3.2 Model training

The deep learning model was trained on 40 data sets. Another 6 data sets were used for model validation to monitor the training process and to determine the best checkpoint. Each data set contained 60 single‐view acquisitions from different angles, generating 2400 and 360 samples for the model training and validation, respectively. Note that a sample refers to an image pair consisting of the single‐view image and the corresponding full‐view image. These data sets were acquired with an electric field intensity ranging from 1200 to 2000 Volts/cm.

In the training process, single‐view images were fed into the deep learning model, whose weights were optimized by minimizing the loss between the predicted and the ground truth full‐view images. The loss function was structural dissimilarity.^24^ The optimizer was “Adam”^34^ with a learning rate of 0.0007. The batch size was 1.

### 3.3 Model evaluation

#### 3.3.1 Evaluation of the enhancement performance

Ten data sets (containing 600 samples, acquired with an electric field intensity of 2400 Volts/cm), excluded from the training and validation datasets, were used for the model testing. The electroacoustic data acquisition and reconstruction followed the process described in section 3.1. The single‐view image was fed into the trained model for enhancement, and was then compared to the corresponding ground truth image reconstructed using full‐view measurements.

#### 3.3.2 Evaluation metrics

Testing results were evaluated both qualitatively and qualitatively using RMSE, peak‐signal‐to‐noise‐ratio (PSNR), structural similarity index matrix (SSIM), and the iso‐pressure line DICE coefficients.

## 4 RESULTS

### 4.1 Pressure map distortion correction

**FIGURE 4:**
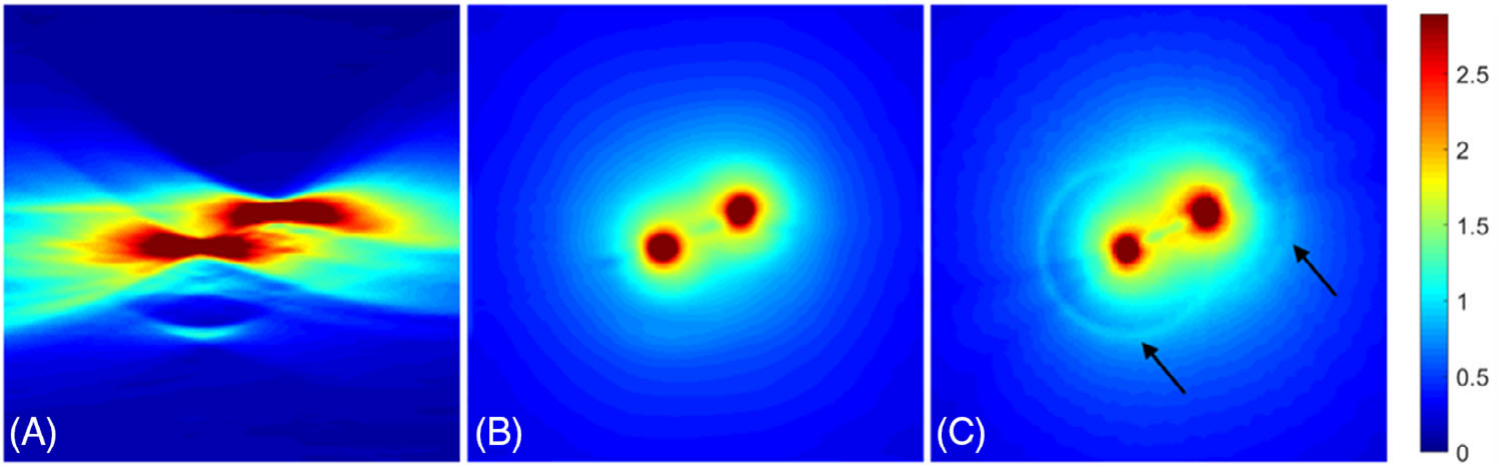
Pressure map of a representative case in the testing dataset. (A) is the pressure map reconstructed from a single‐view linear array's measurement using the back‐projected algorithm. (B) is the pressure map enhanced by the deep learning model. (C) is the ground truth pressure map. Black arrows in (C) indicate the echo artifacts between electrodes. The ‘jet’ color map is used as shown by the color bar in the right.

**FIGURE 5:**
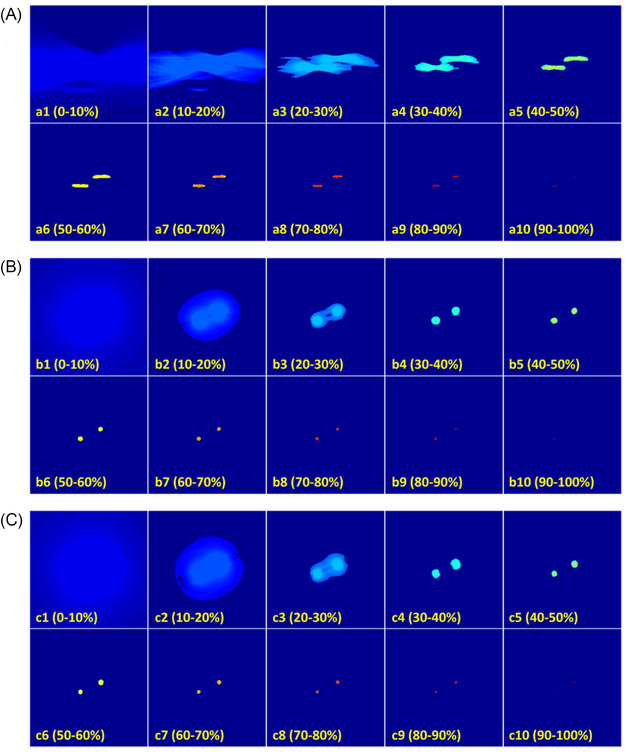
(1‐10) are the images within corresponding value ranges for (A) back‐projected linear‐array reconstruction, (B) deep learning‐enhanced linear‐array reconstruction, and (C) ground truth full‐view reconstruction, respectively. The ‘jet’ color map is used for (A‐C) where red indicates high intensities and blue low.

Figure 4 shows a representative case in the testing dataset. The images are the pressure maps reconstructed in the linear array scanning plane shown in Figure 3. They reflect the electricity energy deposition around two electrodes (shown in the center of the dark red regions). The intensity changes (from red to blue) demonstrate the deposited energy falloff, which is caused by the electric field falloff. Artifacts indicated by black arrows are caused by echoes between electrodes. Due to the single‐view measurement of the linear array, pressure map reconstructed by the back‐projected algorithm showed severe distortions, in which the electric field distribution can hardly be distinguished from artifacts. The proposed method considerably improved the image quality by effectively correcting the distortions and restoring the electricity field distribution. The pressure shape exhibited substantial concordance with the full‐view ground truth. Figure 5 shows the distribution of the electricity field with isolines, further confirming the efficacy of the proposed method in restoring structures from the single‐view measurements.

Table 1 shows the quantitative metrics of the pressures enhanced by the proposed method. Results were calculated using all 600 testing samples. Compared to the conventional back‐projected algorithm, the model‐enhanced images showed substantially lower intensity errors (indicated by lower RMSE), higher SNR (indicated by higher PSNR), higher structural similarity (indicated by higher SSIM), and higher structural shape accuracy (indicated by higher DICEs). Results showed high agreement between the enhanced and the full‐view reconstructions. These quantitative results further confirmed the effectiveness of the deep learning model in restoring image information from the single‐view measurements.

**TABLE 1:** Quantitative analysis. Metrics were calculated using all the testing samples.

Metric	Value	
	Single View	Enhanced
RMSE	0.0821 ± 0.0038	**0.0180 ± 0.0044**
PSNR	21.721 ± 0.4024	**35.155 ± 2.2468**
SSIM	0.3835 ± 0.2892	**0.9420 ± 0.0551**
DICE (10%)	0.3146 ± 0.0751	**0.8022 ± 0.0949**
DICE (20%)	0.2899 ± 0.0453	**0.8565 ± 0.0399**
DICE (30%)	0.3343 ± 0.0404	**0.8497 ± 0.0524**
DICE (40%)	0.3682 ± 0.0483	**0.8111 ± 0.0705**
DICE (50%)	0.4011 ± 0.0476	**0.7625 ± 0.0876**
DICE (60%)	0.4219 ± 0.0386	**0.6962 ± 0.1109**
DICE (70%)	0.4022 ± 0.0464	**0.5886 ± 0.1321**

Intensities of pressure and dose maps are normalized to [0, 1] to calculate the metrics.

^*^Numbers in the table are expressed as mean ± standard deviation.

### 4.2 Runtime

The proposed deep learning model was developed using the Keras framework with the Tensorflow backend. The model training, validation, and testing were performed on a NVIDIA Titan RTX (24GB memory) GPU. The enhancement takes 0.016 seconds for an image of dimensions 256×256.

## 5 DISCUSSION

In this study, we proposed a deep learning‐based method, which is effective in correcting the distortions in the linear array‐based EAT images. In addition, the entire workflow is fully automatic and highly efficient.

Similar studies^31^, ^35^, ^36^, ^37^ have been conducted to employ deep learning models to correct the single‐view distortions using simulated acoustic data. Despite the encouraging results, their performance can be compromised in an experimental environment. To bridge the gap between simulation and experiments, in this study, we fully trained the deep learning model using experimental data in a supervised method. A major challenge for this training strategy is to acquire the paired single‐view and full‐view reconstruction of the same electric field, for which we designed a rotating platform to enable full‐view acquisition using a linear array. Data acquisition can be time‐consuming during model training. However, once the model is trained, it can be used to enhance EAT images measured by a linear array from a single view in nearly real‐time.

Due to the limitation of devices in our lab, the applied electric field intensity is lower than typical electroporation treatment. Results indicated that the model trained using lower voltages (1200 ‐ 2000 Volts/cm) was able to accurately enhance single‐view EAT at higher voltages (2400 Volts/cm), demonstrating the method's generalizability across voltages.

For the first time, we acquired high‐quality EAT imaging from a single linear array's measurement in an experimental environment, which can considerably improve EAT's clinical utility in real‐time treatment monitoring. However, there are some limitations in this study. First, electric energy was deposited in a homogeneous medium (water tank filled with the dilute sodium chloride solution), which did not consider the heterogeneity of human tissues. Besides, relatively simple electrode arrangements were adopted in this study. Nonetheless, this is the first pilot study demonstrating the efficacy of deep learning to predict high‐quality, accurate EAT images using only a single‐view image acquired with a linear array in an experimental environment. In future studies, more complicated experimental setups are warranted to further evaluate this novel technique.

## 6 CONCLUSION

This preliminary study demonstrated that the proposed deep learning‐based method is effective and efficient in acquiring high‐quality EAT using a single linear array in an experimental environment. This innovation bridges the gap between simulation and real‐world application, showing great promise to improve EAT's clinical utility in real‐time monitoring for electroporation treatment.

## AUTHOR CONTRIBUTIONS

Dr. Lei Ren and Dr. Liangzhong Xiang are the corresponding authors who supervised and instructed the entire project.

Zhuoran Jiang led the deep learning‐based method development; Yifei Xu led the electroacoustic data acquisition. Zhuoran Jiang and Yifei Xu designed the workflows for deep learning model training and evaluation, and analyzed the testing results. Leshan Sun provided support for the linear array‐based acoustic image reconstruction; Zhuoran Jiang developed the algorithm for full‐view image reconstruction. Shreyas Srinivasan helped Yifei Xu with the electroacoustic data acquisition. Dr. Q Jackie Wu provided support for the experiment and study conduction.

All the authors contributed to the manuscript and approved the publication of this study.

## CONFLICTS OF INTEREST STATEMENTS

All authors declare that they have no known conflicts of interest in terms of competing financial interests or personal relationships that could have an influence or are relevant to the work reported in this paper.

## ETHICS STATEMENT

Not applicable.

## Data Availability

The experimental electroacoustic data and source code will be available upon request.
